# Orexin neurons mediate temptation-resistant voluntary exercise

**DOI:** 10.1038/s41593-024-01696-2

**Published:** 2024-08-06

**Authors:** Alexander L. Tesmer, Xinyang Li, Eva Bracey, Cyra Schmandt, Rafael Polania, Daria Peleg-Raibstein, Denis Burdakov

**Affiliations:** Neurobehavioural Dynamics Laboratory, Department of Health Sciences and Technology, Eidgenössische Technische Hochschule Zürich, Schwerzenbach, Switzerland

**Keywords:** Decision, Hypothalamus

## Abstract

Despite the well-known health benefits of physical activity, many people underexercise; what drives the prioritization of exercise over alternative options is unclear. We developed a task that enabled us to study how mice freely and rapidly alternate between wheel running and other voluntary activities, such as eating palatable food. When multiple alternatives were available, mice chose to spend a substantial amount of time wheel running without any extrinsic reward and maintained this behavior even when palatable food was added as an option. Causal manipulations and correlative analyses of appetitive and consummatory processes revealed this preference for wheel running to be instantiated by hypothalamic hypocretin/orexin neurons (HONs). The effect of HON manipulations on wheel running and eating was strongly context-dependent, being the largest in the scenario where both options were available. Overall, these data suggest that HON activity enables an eat–run arbitration that results in choosing exercise over food.

## Abstract (web summary)

What makes the brain maintain voluntary exercise despite attractive alternative options such as eating? Tesmer et al. show that orexin/hypocretin neurons are crucial for implementing the underlying valuation of eating versus running in mice.

## Main

There is an overwhelming agreement in the scientific literature and global health guidelines that physical exercise has acute and chronic benefits for diverse aspects of health^1–6^. While some people choose to exercise over other social and recreational activities^7^, many people underexercise and overeat highly palatable food (HPF) that is widely available in many societies^8–10^. This is considered a global health problem^1,11,12^.

Classic and recent studies implicate the lateral hypothalamus as important for the motivation to move^13–15^. However, whether lateral hypothalamic neurons regulate attraction to, and engagement in, voluntary exercise, and whether this depends on the availability of alternative activities, is unclear. Lateral hypothalamic hypocretin/orexin neurons (HONs) are thought to regulate both food consumption and energy balance over chronic timescales^16–22^. HONs produce and release the peptide neurotransmitters orexins/hypocretins^16,19,23^. These transmitters activate specific G-protein-coupled receptors that are distributed brain-wide, and serve as targets for an increasing number of human-approved pharmaceuticals^24–29^. However, the acute role of HONs in eating is unclear^30,31^, and whether they are involved in rapid arbitration between HPF and exercise when multiple choices are available is not known.

One way to investigate voluntary exercise in animal models is to provide an opportunity to choose between exercise, palatable food and other attractive options. In this study, using voluntary wheel running in mice as a well-established model for human health-beneficial voluntary exercise^32,33^, we perform experiments aimed at assessing the role of HONs in temptation-resistant exercise in multiple choice scenarios that evoke acute decisions of whether to exercise or engage in other pursuits. We then relate our findings to the frameworks of the appetitive (‘approach’) and consummatory (‘engagement’) phases of eating and exercising, and provide a mechanistic neuroeconomic account of preference toward exercise. Our results provide evidence that HONs are essential for maintaining voluntary exercise when a palatable food choice is available, and that HONs implement this by a context-specific eat–run valuation rather than by separate control of eating or running.

## Results

### Temptation-resistant exercise requires the orexin system

**Fig. 1:**
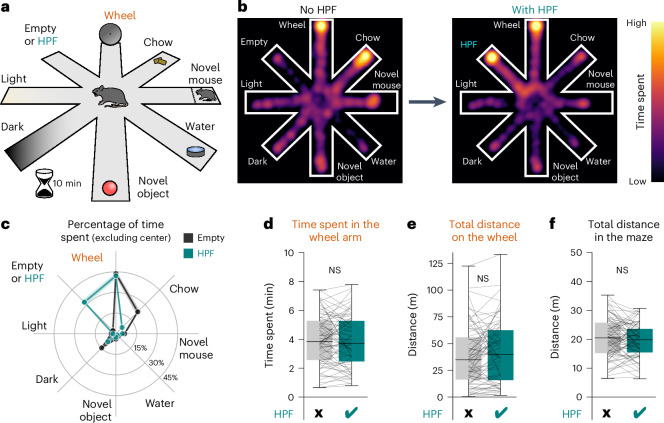
TRVE in mice. **a**, Mice (*n* = 71) explored an eight-arm maze containing distinct alternatives at the end of each arm. Mouse location was video-tracked over a 10-min period. **b**, Heatmaps of an example mouse displaying a shift in time spent in the chow arm (left) toward the HPF, when available (right). **c**, In the maze version lacking the HPF option (black), mice spent the most time in the wheel and chow arms. In the maze version with the HPF option available (teal), mice spent the most time in the wheel and HPF arms. The lines represent the means; the shaded regions represent the s.e.m. of *n* = 71 mice. **d**, Total time spent in the wheel arm in the absence and presence of the HPF option (paired *t*-test: *t*_70_ = −0.683, *P* = 0.497, *n* = 71 mice). **e**, Total distance traveled on the wheel in the absence and presence of the HPF option (paired *t*-test: *t*_70_ = 1.514, *P* = 0.134, *n* = 71 mice). **f**, Total distance run in the *xy* plane of the maze outside the wheel in the absence and presence of the HPF option (paired *t*-test: *t*_70_ = −1.147, *P* = 0.256, *n* = 71 mice). NS, not significant. Box plots: the center line is the median, the box edges are the top and bottom quartiles, the whiskers are minimum and maximum. Source data

**Extended Data Fig. 1:**
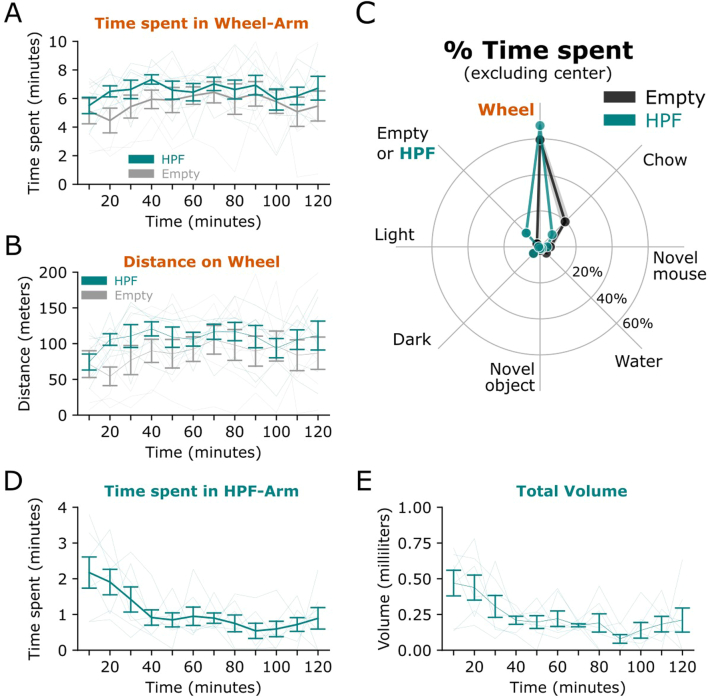
TRVE persists for hours. **a** Time spent in the wheel-arm when HPF was not available (grey) or available (teal). Each point represents occupancy in 10-minute time-bins, n = 7 mice. Thick lines represent mean and SEM. Faint lines represent individual mice. **b** Distance traveled on the running-wheel in 10-minute time bins, n = 7 mice. Thick lines represent mean and SEM. Faint lines represent individual mice. **c** Mice spent the most time in the wheel arm regardless of whether the HPF was available (teal) or not (black). Shaded regions represent SEM error bars, n = 7 mice. **d** Same as A, Time spent in the HPF-arm in 10-minute time bins, n = 7 mice. Thick lines represent mean and SEM. Faint lines represent individual mice. **e** Same as A, Volume of HPF consumed in 10-minute time bins, n = 7 mice. Thick lines represent mean and SEM. Faint lines represent individual mice. Source data

We placed mice in the center of an eight-arm maze; the mouse could freely choose between arms (Fig. 1a,b). Each arm contained one option, all placed equidistant from the center. The options included a running wheel, a novel (i.e., unfamiliar) object, a novel mouse, water, a light area, a dark area and chow; one arm was either left empty or contained HPF. When the food option was limited to standard chow, mice chose to split most of their time between the running wheel and chow (black trace, Fig. 1c). When HPF was added to the alternatives, mice substantially reduced their time at chow (teal trace, Fig. 1c). Strikingly, however, running wheel occupation and usage was unaltered in the presence of HPF (Fig. 1d,e), as was total distance traveled in the maze outside the running wheel (Fig. 1f). Sessions were deliberately kept short (10 min) in these experiments to capture the initial decision-making processes while minimizing the confounding effects of fatigue and satiation; however, we also found that the running wheel preference persisted if the duration was extended to hours (Extended Data Fig. 1). Overall, these observations identify a mouse model for voluntary exercise-like activity that is resistant to HPF ‘temptation’, henceforth termed temptation-resistant voluntary exercise (TRVE).

**Fig. 2:**
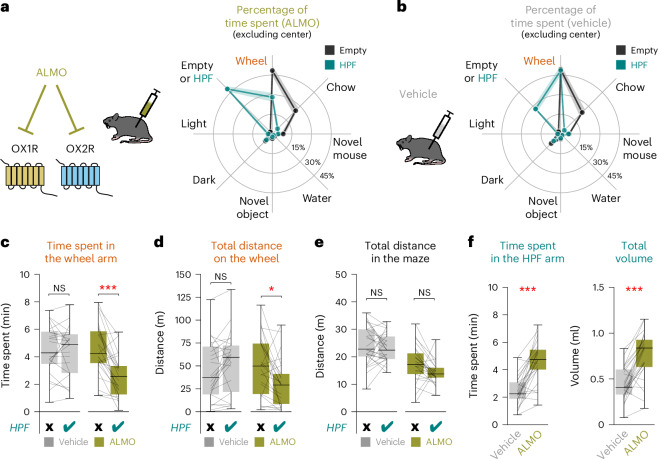
TRVE requires orexin receptor signaling. **a**, Mice were injected with 30 mg kg^−1^ ALMO intraperitoneally 40 min before being placed into the maze for 10 min. Mice spent most of the time in the wheel and chow arms when the HPF option was not available (black). Mice spent most of the time in the wheel and HPF arms when the HPF option was available (teal); note that time spent in wheel arm was lower when HPF was available. The lines represent the mean and the shaded regions represent the s.e.m. of *n* = 25 mice. **b**, Same as in **a** but for mice injected with vehicle (control mice). Note that the addition of the HPF option did not reduce the time spent on the wheel arm. The lines represent the mean and the shaded regions represent the s.e.m. of *n* = 25 mice. **c**, Effect of HPF and ALMO on total time spent in the wheel arm in *n* = 25 mice (repeated measures analysis of variance (ANOVA): drug × HPF interaction *F*_1,24_ = 22.577, *P* = 0.00008). Bonferroni-corrected paired *t*-test: vehicle *t*_24_ = −0.123, *P* = 1.000; ALMO *t*_24_ = −5.197, *P* = 0.00005. **d**, Total distance traveled on the running wheel in *n* = 25 mice (repeated measures ANOVA: drug × HPF interaction *F*_1,24_ = 18.069, *P* = 0.0003). Bonferroni-corrected paired *t*-test: vehicle *t*_24_ = 2.002, *P* = 0.113; ALMO *t*_24_ = −2.853, *P* = 0.018. **e**, Total distance run in the maze in *n* = 25 mice (repeated measures ANOVA: drug × HPF interaction *F*_1,24_ = 0.212, *P* = 0.649). Bonferroni-corrected paired *t*-test: vehicle *t*_24_ = −1.764, *P* = 0.181; ALMO *t*_24_ = −2.209, *P* = 0.074. **f**, ALMO-induced changes in *n* = 25 mice. Left, time spent in the HPF arm (paired *t*-test: *t*_24_ = 6.578, *P* = 8 × 10^−7^). Right, HPF consumption (paired *t*-test: *t*_24_ = 6.226, *P* = 2 × 10^−6^). *P < 0.05, ***P < 0.001. Box plots: the center line is the median, the box edges are the top and bottom quartiles, the whiskers are the minimum and maximum. Source data

**Extended Data Fig. 2:**
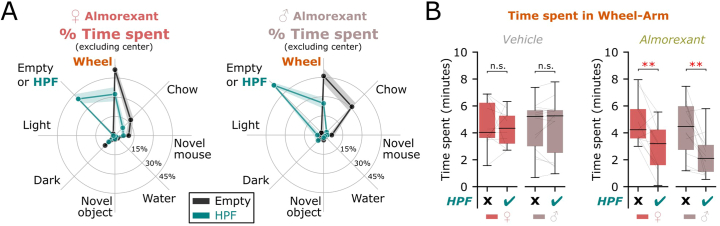
Orexin receptor-dependent TRVE in males and females. **a** Almorexant-injected female (n = 11, left) and male (n = 14, right) mice displayed the most time spent in the wheel arm and the chow arm when the HPF option was not available (black). Both sexes displayed the most time spent in the wheel arm and the HPF arm when the HPF option was available (teal). Lines represent mean and SEM. **b** Left: Effect of sex and HPF on total time spent in the wheel-arm in vehicle-injected (mixed ANOVA: sex × HPF interaction *F*_*1,23*_ = 0.463, p = 0.503). Post-hoc comparisons using Bonferroni-corrected paired *t*-tests revealed no significant differences in n = 11 female mice (*t*_*10*_ = 0.432, p = 0.675) nor n = 14 male mice (*t*_*13*_ = 0.627, p = 0.544). Right: Effect of sex and HPF on total time spent in the wheel-arm in almorexant-injected (mixed ANOVA: sex × HPF interaction *F*_*1,23*_ = 0.069, p = 0.796). Post-hoc comparisons using Bonferroni-corrected paired *t*-tests revealed significant differences in n = 11 female mice (*t*_*10*_ = −3.644, p = 0.009) and n = 14 male mice (*t*_*13*_ = −3.666, p = 0.006). *p < 0.05, **p < 0.01, ***p < 0.001 and n.s., not significant by two-tailed tests. Box plots: center line is median, box edges are top and bottom quartiles, whiskers are minimum and maximum. Source data

**Extended Data Fig. 3:**
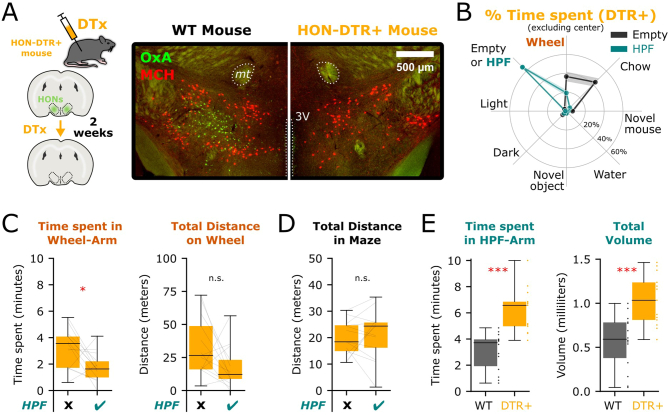
Effect of HON ablation on TRVE. **a** To ablate HONs, male HON-DTR mice were injected with diphtheria toxin >2 weeks before experiments. Brain slice histology of a wild-type mouse (WT, left, image representative of 10 mice) and a HON-ablated mouse (DTR+, right, image representative of 10 mice). OxA = orexin-A, MCH = melanin-concentrating hormone (control staining, to show lack of loss of MCH and selective loss of OxA), 3V = 3^rd^ ventricle, mt = mammillothalamic tract. **b** Mice displayed the most time spent in the wheel arm and the chow arm in the maze version that did not have an HPF option (black); when that option was available (teal), mice spent the most time spent in the wheel arm and the HPF arm. Note that time spent in the wheel arm was reduced when the HPF option was added. Lines represent mean and shaded areas represent SEM of n = 13 male mice. **c** Left: Effect of HPF on total time spent in the wheel-arm in n = 13 HON-ablated mice (paired *t*-test: *t*_*12*_ = −2.667, p = 0.021). Right: effect of HPF on total distance traveled on the wheel in n = 13 HON-ablated mice (paired *t*-test: *t*_*12*_ = −1.966, p = 0.073). **d** Effect of HPF on total distance run in the maze in n = 13 HON-ablated mice (paired *t*-test: *t*_*12*_ = −1.181, adjusted p = 0.260). **e** Left: Effect of HON ablation on total time spent in the HPF arm in n = 13 mice (unpaired *t*-test: *t*_*25*_ = 5.130, adjusted p = 0.00003). Right: Effect of HON ablation on total volume of HPF consumed in n = 13 mice (unpaired *t*-test: *t*_*25*_ = 4.060, p = 0.0004). *p < 0.05, **p < 0.01, ***p < 0.001 and n.s., not significant by two-tailed tests. Box plots: center line is median, box edges are top and bottom quartiles, whiskers are minimum and maximum. Source data

Next, we tested how disruption of orexin neuropeptide signaling affects TRVE. We found that intraperitoneal injection of the orexin receptor antagonist almorexant (ALMO), which blocks the two orexin receptors that both exist in mice and humans^34^, 40 min before the task abolished TRVE (Fig. 2). Similar effects occurred in males and females (Extended Data Fig. 2). Thus, all other analyses included both sexes (Extended Data Table 1). In the presence of ALMO, HPF addition to the maze significantly reduced both the time spent in the running wheel arm (Fig. 2a–c) and running wheel use (Fig. 2d), but not the total distance traveled outside the running wheel (Fig. 2e). This indicates that when orexin receptors were antagonized, the presence of HPF selectively reduced the ‘attractiveness’ and use of the running wheel, rather than affecting general locomotion. Under ALMO, the reduced running wheel use was associated with increased HPF behaviors (that is, time spent in the HPF area and amount of HPF consumed; Fig. 2f). Deletion of HONs, through the previously validated HON-specific orexin-diphtheria (DTR) cell ablation model^31,35^, also disrupted TRVE (Extended Data Fig. 3). Overall, these data indicate that HONs, and orexin receptor signaling in particular, are necessary for TRVE.

### Mechanisms of orexin-dependent TRVE

**Fig. 3:**
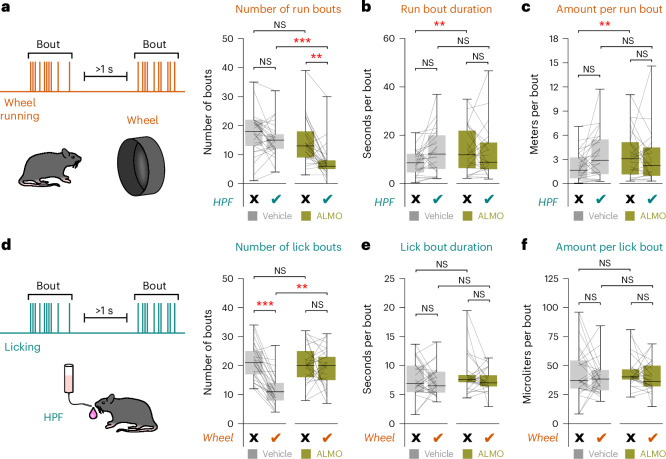
Behavioral microstructure underlying TRVE and the effects of orexin receptor blockade. **a**, Left, run bout definition. Right, effect of HPF and ALMO number of run bouts in *n* = 25 mice (repeated measures ANOVA: drug × HPF interaction *F*_1,24_ = 8.334, *P* = 0.008). Bonferroni-corrected Wilcoxon signed-rank test: vehicle *T* = 108.5, *P* = 0.625; ALMO *T* = 18.5, *P* = 0.001; no HPF *T* = 89.5, *P* = 0.333; with HPF *T* = 10.5, *P* = 0.00001. **b**, Run bout duration in *n* = 24 mice (repeated measures ANOVA: drug × HPF interaction *F*_1,23_ = 6.092, *P* = 0.021). Bonferroni-corrected paired *t*-test: vehicle *t*_23_ = 2.620, *P* = 0.061; ALMO *t*_23_ = −0.370, *P* = 1.000; no HPF *t*_23_ = 3.540, *P* = 0.007; with HPF *t*_23_ = −0.268, *P* = 1.000. **c**, Meters run per bout in *n* = 24 mice (repeated measures ANOVA: drug × HPF interaction *F*_1,23_ = 8.647, *P* = 0.007). Bonferroni-corrected paired *t*-test: vehicle: *t*_23_ = 2.607, *P* = 0.063; ALMO *t*_23_ = −0.628, *P* = 1.000; no HPF *t*_23_ = 3.522, *P* = 0.007; with HPF *t*_23_ = −0.870, *P* = 1.000. **d**, Left, lick bout definition. Right, effect of wheel and ALMO on the number of lick bouts in *n* = 25 mice (repeated measures ANOVA: drug × wheel interaction *F*_1,24_ = 18.797, *P* = 0.0002). Bonferroni-corrected Wilcoxon signed-rank test: vehicle *T* = 2.5, *P* = 0.0001; ALMO *T* = 141, *P* = 1.000; no wheel *T* = 150, *P* = 1.000; with wheel *T* = 36.5, *P* = 0.001. **e**, Lick bout duration in *n* = 25 mice (repeated measures ANOVA: drug × wheel interaction *F*_1,24_ = 0.402, *P* = 0.532). Bonferroni-corrected paired *t*-test: vehicle *t*_24_ = −1.003, *P* = 1.000; ALMO: paired *t*-test: *t*_24_ = −1.405, *P* = 0.691; no wheel *t*_24_ = 1.076, *P* = 1.000; with wheel *t*_24_ = 0.613, *P* = 1.000. **f**, Volume per lick bout in *n* = 25 mice (repeated measures ANOVA: drug × wheel interaction *F*_1,24_ = 0.001, *P* = 0.970). Bonferroni-corrected paired *t*-test: vehicle *t*_24_ = −0.873, *P* = 1.000; ALMO *t*_24_ = −0.993, *P* = 1.000; no wheel *t*_24_ = −0.017, *P* = 1.000; with wheel *t*_24_ = 0.052, *P* = 1.000. **P < 0.01, ***P < 0.001. Box plots: the center line is the median, the box edges are the top and bottom quartiles, the whiskers are the minimum and maximum. Source data

**Extended Data Fig. 4:**
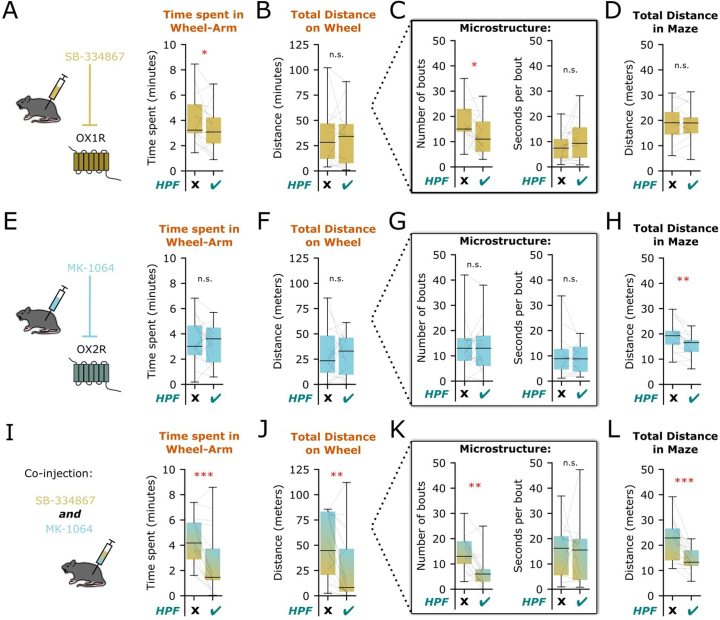
Roles of orexin receptor subtypes in TRVE. **a** Subjects (n = 17) were injected with an OX1R antagonist: SB-334867. Note a disruption of TRVE quantified by time spent in wheel-arm (paired *t*-test: *t*_*16*_ = −2.211, p = 0.042). **b** Effect of HPF on wheel-distance in n = 17 mice (paired *t*-test: *t*_*16*_ = −0.414, p = 0.684). **c** Effect of HPF on wheel-running microstructure in n = 17 mice quantified via number of bouts (Wilcoxon test: *D* = 24, p = 0.011) and bout duration (paired *t*-test: *t*_*16*_ = 1.585, p = 0.133). **d** Effect of HPF on total distance run in the maze in n = 17 mice (paired *t*-test: *t*_*16*_ = 0.350, p = 0.731). **e** Subjects (n = 17) were injected with an OX2R antagonist: MK-1064. Note no disruption of TRVE quantified by time spent in wheel-arm (paired *t*-test: *t*_*16*_ = −0.493, p = 0.629). **f** Effect of HPF on wheel-distance in n = 17 mice (paired *t*-test: *t*_*16*_ = −0.040, p = 0.969). **g** Effect of HPF on wheel-running microstructure in n = 17 mice quantified via number of bouts (Wilcoxon test: *D* = 68, p = 1.000) and bout duration (paired *t*-test: *t*_*14*_ = −0.799, p = 0.438). **h** Effect of HPF on total distance run in the maze in n = 17 mice (paired *t*-test: *t*_*16*_ = 3.498, p = 0.003). **i** Subjects (n = 17) were co-injected with a mixture of both SB-334867 and MK-1064. Note a disruption of TRVE quantified by time spent in wheel-arm (paired *t*-test: *t*_*16*_ = −4.207, p = 0.0007). **j** Effect of HPF on wheel-distance in n = 17 mice (paired *t*-test: *t*_*16*_ = −3.013, p = 0.008). **k** Effect of HPF on wheel-running microstructure in n = 17 mice quantified via number of bouts (Wilcoxon test: *D* = 15.5, p = 0.003) and bout duration (paired *t*-test: *t*_*14*_ = −0.136, p = 0.894). **l** Effect of HPF on total distance run in the maze in n = 17 mice (paired *t*-test: *t*_*16*_ = 4.356, p = 0.0005). *p < 0.05, **p < 0.01, ***p < 0.001 and n.s., not significant by two-tailed tests. Box plots: center line is median, box edges are top and bottom quartiles, whiskers are minimum and maximum. Source data

To examine the behavioral microstructure mechanisms underlying orexin-dependent TRVE, we quantified the behavioral bout number and duration. For eating and running, these metrics reflect the dissociable processes of behavioral initiation and maintenance, respectively^36,37^. ALMO-evoked disruption of TRVE reduced the running bout number but not the duration or the distance run per bout (Fig. 3a–c); and increased the number of HPF eating bouts but not their duration or the volume eaten per bout (Fig. 3d–f). This suggests that, in the arena with both running wheel and HPF options available, endogenous orexin receptor activity normally promotes the initiation of wheel running and limits the initiation of HPF eating. Of note, the orexin type-1 receptor antagonist SB-334867 (ref. ^38^) reproduced the ALMO effect to some extent, whereas the orexin type-2 receptor antagonist MK-1064 (ref. ^39^) had little effect on TRVE but reduced locomotion outside the running wheel (Extended Data Fig. 4a–h). However, applying SB-334867 and MK-1064 together produced a supra-additive effect on TRVE equivalent to that of ALMO (Extended Data Fig. 4i–k), suggesting orexin receptor synergy in regulating exercise initiation in TRVE.

**Fig. 4:**
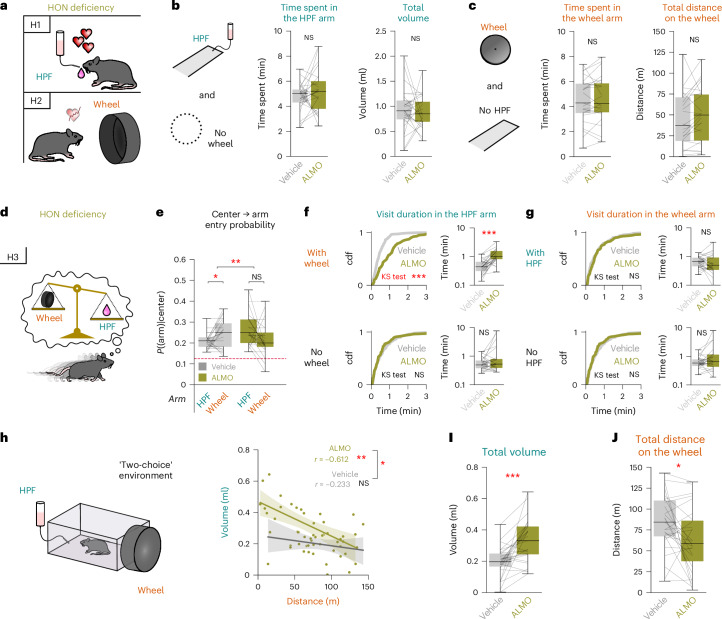
Disentangling the decision-making processes underlying orexin receptor-dependent TRVE. **a**, Schematic of two initial hypotheses. **b**, Effect of ALMO when the wheel was unavailable in *n* = 25 mice. Bonferroni-corrected paired *t*-test: left *t*_24_ = 1.164, *P* = 0.512; right *t*_24_ = −0.303, *P* = 1.000. **c**, Effect of ALMO when HPF was unavailable in *n* = 25 mice. Bonferroni-corrected paired *t*-test: left *t*_24_ = 0.417, *P* = 1.000; right *t*_24_ = 1.729, *P* = 0.193. **d**, Schematic of an alternative hypothesis. **e**, Effect of ALMO on arm entry probability in *n* = 25 mice (repeated measures ANOVA: drug × arm interaction *F*_1,24_ = 10.460, *P* = 0.004). Bonferroni-corrected paired *t*-test: vehicle *t*_24_ = 2.566, *P* = 0.034; ALMO *t*_24_ = −2.246, *P* = 0.068. The dashed line depicts random choice. **f**, Left, cumulative plot of the duration of all HPF arm visits from *n* = 25 mice when the running wheel was available (top) or unavailable (bottom). Bonferroni-corrected two-sample Kolmogorov–Smirnov (KS) test: top *D* = 0.100, *P* = 0.475; bottom *D* = 0.402, *P* = 1 × 10^−8^. Right, corresponding per-mouse-averaged visit duration. Bonferroni-corrected paired *t*-test: top *t*_24_ = 6.094, *P* = 3 × 10^−6^; bottom *t*_24_ = 1.891, *P* = 0.071. cdf, cumulative density function. **g**, Left, cumulative plot of the duration of all wheel arm visits from *n* = 25 mice when HPF was available (top) or unavailable (bottom). Bonferroni-corrected two-sample KS test: top *D* = 0.095, *P* = 1.000; bottom *D* = 0.107, *P* = 0. 870. Right, corresponding per-mouse-averaged visit duration. Bonferroni-corrected paired *t*-test: top *t*_24_ = 0.294, *P* = 1.000; bottom *t*_24_ = 1.674, *P* = 0.214. **h**, Left, schematic of the ‘two-choice’ experiment. Right, correlation between HPF and running wheel use in *n* = 27 mice (the shaded regions represent the 95% confidence intervals (CIs)). ALMO: Pearson’s *r* = −0.612; Wald test, corrected *P* = 0.001. Vehicle: Pearson’s *r* = −0.233; Wald test, corrected *P* = 0.486. Comparison was carried out with a Fisher’s test, *z* = −2.329, *P* = 0.019. **i**, HPF consumption in the ‘two-choice’ experiment (paired *t*-test: *t*_26_ = 4.627, *P* < 0.001, *n* = 27 mice). No drug × sex interaction via mixed ANOVA: *F*_1,25_ = 0.936, *P* = 0.3425. **j**, Distance run on the wheel in the ‘two-choice’ experiment (paired *t*-test: *t*_26_ = −2.611, *P* = 0.015, *n* = 27 mice). No drug × sex interaction via mixed ANOVA: *F*_1,25_ = 0.622, *P* = 0.438. *P < 0.05, **P < 0.01, ***P < 0.001. Box plots: the center line is the median, the box edges are the top and bottom quartiles, the whiskers are the minimum and maximum. Source data

Next, we examined the decision-making processes underlying TRVE. It is currently thought that orexin peptides increase locomotion and modulate eating^16,40^ (Fig. 4a). However, when the HPF but not the running wheel was present in the maze, we found that ALMO affected neither appetitive nor consummatory processes related to HPF (quantified in this study as time spent in the HPF area and the HPF volume consumed, respectively: Fig. 4b). Similarly, when the running wheel but not the HPF was present, ALMO affected neither appetitive nor consummatory processes related to the running wheel (time spent in the running wheel area and distance covered on the running wheel, respectively; Fig. 4c). Note that ALMO strongly reduced the two running wheel area occupancy and use when both running wheel and HPF were available (Fig. 2c,d). These findings indicate that, in the multiple choice maze apparatus, TRVE cannot be explained by orexin-dependent modulation of running wheel-directed or HPF-directed processes in isolation.

An alternative hypothesis is that HONs shape value comparisons between running wheel and HPF (Fig. 4d). This predicts that ALMO would affect running wheel and HPF use only in a scenario in which both are available. To look at the behavioral components of such a valuation, we first examined how mice decided to enter either the running wheel or HPF arm from a neutral zone (the center area). We found that control (vehicle-injected) mice were more likely to enter the running wheel arm than the HPF arm, whereas ALMO-injected mice were not (Fig. 4e). These results suggest that HONs may facilitate decision-making, directing the mice toward the running wheel over the HPF. However, given that the HPF versus running wheel entry probabilities were similar under ALMO (Fig. 4e), what then explains the reduced wheel running in ALMO-injected mice? Value-based choices include not only the decision to enter a maze arm, but also the decision to leave an arm. Quantification of visit duration in each arm revealed that ALMO increased the average visit duration to the HPF arm when the running wheel was available but had no effect when the running wheel was not available (Fig. 4f). ALMO did not modulate running wheel visit durations, regardless of whether the HPF was available (Fig. 4g). This suggests that HONs promote TRVE by decreasing HPF visit duration when the running wheel is available.

Consummatory behaviors typically involve value comparisons. To probe the effects of HONs on the use of HPF versus running wheel more directly, we placed mice into a cage in which both running wheel and HPF were available in close proximity, thus minimizing the influence of potential appetitive place preferences (Fig. 4h). We found that in ALMO-injected and vehicle-injected (control) mice, the correlations between the volume of HPF consumed and distance traveled on the running wheel (Fig. 4h) were significantly different (Fisher’s exact test, *z* = −2.329, *P* = 0.019). In control mice, there was no significant relationship between running and eating, whereas in ALMO-injected mice eating was inversely proportional to running (Fig. 4h–j), such that mice that ran less consumed more HPF (Pearson’s *r* = −0.612, *P* = 0.001). Overall, these data indicate that the function of HONs in the acute TRVE context cannot be explained by their previously proposed roles as promoters of physical activity or of eating, nor by running-induced under-eating (Fig. 4h). Rather, the results suggest that orexin arbitrates between eating and running, without influencing appetitive or consummatory drives toward either.

### HON dynamics and TRVE

**Fig. 5:**
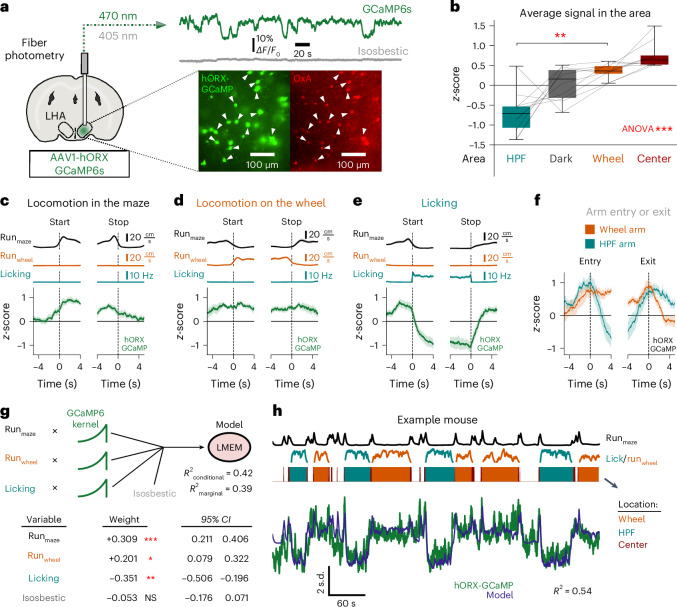
HON dynamics during voluntary behavior in the maze. **a**, Left, targeting schematic of GCaMP6s in HONs. Top right, example trace of activity at 470 nm along with the isosbestic 405-nm reference. Bottom right, example of GCaMP6s localization in an orexin-A (OxA)-stained brain slice (representative example of *n* = 10 mice). The white arrows indicate examples of overlapping fluorescence. LHA, lateral hypothalamic area. **b**, Average HON-specific orexin promoter (hORX)-GCaMP6s signal from *n* = 10 mice in different areas of the maze (repeated measures ANOVA: *F*_3,27_ = 20.110, *P* = 5 × 10^−7^). Note that the average activity in the wheel arm was higher than in the HPF arm (two-tailed paired *t*-test: *t*_9_ = 4.731, *P* = 0.001). Box plots: the center line is the median, the box edges are the top and bottom quartiles, the whiskers are the minimum and maximum. **c**, hORX-GCaMP6s signal (bottom, green) aligned to the start and stop of locomotor bouts in the maze. Mean ± s.e.m. of *n* = 10 mice. **d**, As in **c** but aligned to locomotor bouts on the running wheel. **e**, As in **c** but aligned to bouts of HPF licking. **f**, hORX-GCaMP6s aligned to entry or exit from the wheel (orange) and HPF (teal) arms. Mean ± s.e.m., *n* = 10 mice. **g**, Top, diagram of convolved inputs from *n* = 10 mice into the LMEM. Bottom, table containing the output of the fixed effects (weights) of the model input variables with 95% CIs. The two-tailed *P* values for the fixed effects were derived using the Satterthwaite’s method: Run_Maze_ *t* = 6.500, *P* = 0.0004; Run_Wheel_ *t* = 3.379, *P* = 0.032; Licking *t* = −4.636, *P* = 0.005; Isosbestic *t* = −0.873, *P* = 1.000 after Bonferroni correction. **h**, Top, example of convolved behavioral traces of maze running, licking and wheel running. Middle, the filled bars represent the location. Bottom, hORX-GCaMP6s photometry (green) and model-predicted HON activity (blue). *P < 0.05, **P < 0.01, ***P < 0.001. Source data

**Extended Data Fig. 5:**
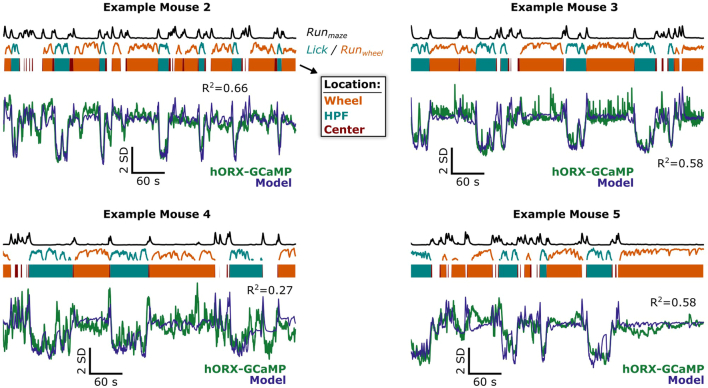
Additional examples of neurobehavioral dynamics data, and model fits. Four additional examples of fitted model performance. See description in Fig. 5.

Do the rapid dynamics of HONs track behavioral choices during TRVE? To probe this, we performed real-time fiber photometry recordings from the lateral hypothalamus of mice selectively expressing the fluorescent neural activity indicator GCaMP6 in HONs (Fig. 5a and Extended Data Fig. 5). In mice behaving inside the maze, the HON signal fluctuated considerably, both as a function of the mouse’s location (Fig. 5b), and during behavioral transitions such as start or stop of locomotion, start or stop of wheel running, and start or stop of licking (Fig. 5c–e). HON activity gradually increased seconds before transition from the maze center to either the running wheel or HPF arm, and before transition from the running wheel or HPF arm to the center (Fig. 5f).

To provide a formal description of the extent to which rapid HON population dynamics report and encode behavioral commitments, we fitted a linear mixed-effects model (LMEM) with the HON signal as the response variable and wheel running, non-wheel locomotion and HPF licking as the input variables (Fig. 5g,h and Extended Data Fig. 5). We found that HON activity could be predicted from the behavioral variables (conditional *R*^2^ = 0.42, marginal *R*^2^ = 0.39; Fig. 5g). Weight estimates showed that the HON signal was negatively related to licking and positively related to wheel running and non-wheel running speed (Fig. 5g, bottom).

**Fig. 6:**
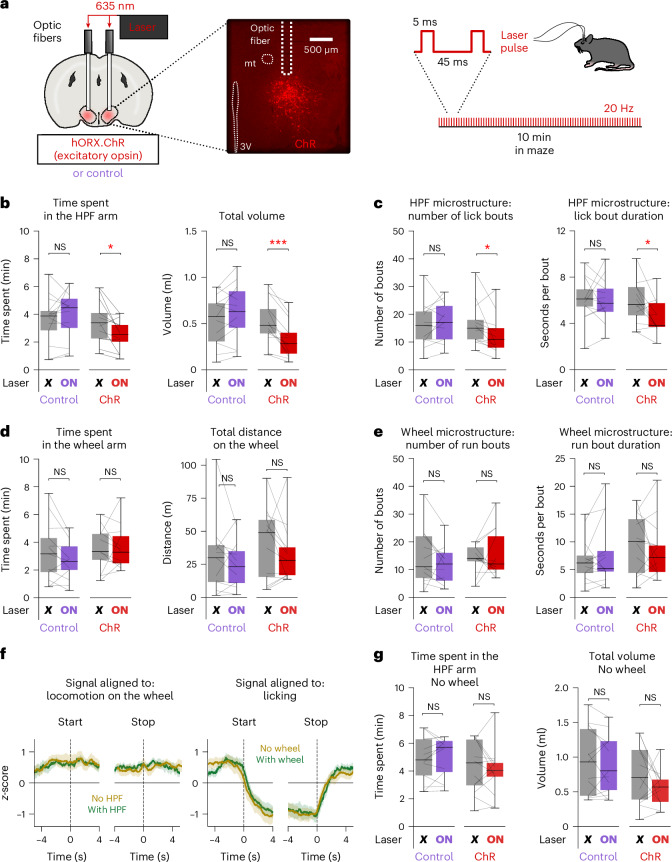
Role of HON dynamics in TRVE. **a**, Left, schematic of HON-targeted ChrimsonR (ChR). Right, example expression of HON-targeted ChR (Methods). 3V, third ventricle; mt, mammillothalamic tract (representative example of *n* = 13 mice). **b**, Left, effect of HON optostimulation on the time spent in the HPF arm. Bonferroni-corrected paired *t*-test: control *n* = 13, *t*_12_ = 0.868, *P* = 0.805; ChR *n* = 13, *t*_12_ = −2.967, *P* = 0.024. Right, same for the HPF volume consumed. Bonferroni-corrected paired *t*-test: control *n* = 13, *t*_12_ = 1.750, *P* = 0.211; ChR *n* = 13, *t*_12_ = −5.296, *P* = 0.0004. **c**, Left, effect of HON optostimulation on the number of lick bouts. Bonferroni-corrected Wilcoxon signed-rank test: control *n* = 13, *D* = 21, *P* = 0.569; ChR *n* = 13, *D* = 13, *P* = 0.043. Right, same but for lick bout duration. Bonferroni-corrected paired *t*-test: control *n* = 13, *t*_12_ = −0.019, *P* = 1.000; ChR *n* = 13, *t*_12_ = 2.642, *P* = 0.043. **d**, Left, effect of HON optostimulation on the time spent in the wheel arm. Bonferroni-corrected paired *t*-test: control *n* = 13, *t*_12_ = −1.875, *P* = 0.171; ChR *n* = 13, *t*_12_ = 0.077, *P* = 1.000. Right, same for the distance run on the wheel. Bonferroni-corrected paired *t*-test: control *n* = 13, *t*_12_ = −0.819, *P* = 0.857; ChR *n* = 13, *t*_12_ = −1.205, *P* = 0.503), **e**, Left, effect of HON optostimulation on the number of wheel run bouts. Bonferroni-corrected Wilcoxon signed-rank test: control *n* = 13, *D* = 18.5, *P* = 0.215; ChR *n* = 13, *D* = 32, *P* = 1.000. Right, same but for run bout duration. Bonferroni-corrected paired *t*-test: control *n* = 13, *t*_12_ = 1.193, *P* = 0.512; ChR *n* = 13, *t*_12_ = −0.863, *P* = 0.810. **f**, Left, HON-GCaMP6s signal at the start and end of the wheel running bouts. Right, same but aligned to the start and end of the HPF licking bouts (green: maze with the running wheel; brown: maze without the running wheel). Mean ± s.e.m. of *n* = 10 mice. **g**, Same as in **b** but in a maze without a wheel. Left, effect of HON optostimulation on the time spent in the HPF arm. Bonferroni-corrected paired *t*-test: control *n* = 13, *t*_12_ = 0.694, *P* = 1.000; ChR *n* = 13, *t*_12_ = −0.739, *P* = 0.948. Right, same but for the HPF volume consumed. Bonferroni-corrected paired *t*-test: control *n* = 13, *t*_12_ = −0.531, *P* = 1.000; ChR *n* = 13, *t*-test: *t*_12_ = −1.463, *P* = 0.339). *P < 0.05, ***P < 0.001. Box plots: the center line is the median, the box edges are the top and bottom quartiles, the whiskers are the minimum and maximum. Source data

To examine whether natural HON activity fluctuations are important for behavior, we induced a constant artificially high state using sustained HON-selective optostimulation (Fig. 6a). Optostimulation reduced the metrics of behaviors that normally coincided with low HON activity (time spent in the HPF arm, total volume of HPF consumed and number and duration of lick bouts; Fig. 6b,c), but did not affect the metrics of running behavior where natural HON activity was already high (Fig. 6d,e). This confirms that natural HON dynamics guide behavior in the choice scenario. Moreover, the HON activity associated with starts and stops of wheel running was similar irrespective of HPF availability, while the HON activity associated with starts and stops of HPF licking was similar irrespective of wheel availability (Fig. 6f), despite the different effects of orexin antagonism in these two scenarios (Figs. 2 and 4a–c). This suggests that the behavioral effect of HON dynamics is context-dependent. In further support of this interpretation, we found that HON optostimulation reduced the time spent in the HPF arm and the volume of HPF consumed when the wheel was available (Fig. 6b), but not when the wheel was unavailable (Fig. 6g). Overall, these data revealed that natural HON activity fluctuations are important for prioritization of exercise over HPF.

## Discussion

The deleterious effects of chronic HPF overconsumption and associated obesity on many neural and cognitive metrics are well documented, but what drives HPF overconsumption in the first place is less understood. In particular, the neural determinants of arbitration between exercising and HPF consumption are unclear. We found causal and correlative evidence that HONs are involved in TRVE. It has been proposed that, owing to presumed food shortage, the brain evolved to prioritize feeding over competing demands, thus producing overconsumption when food is freely available^41,42^. Yet, in a naturalistic, complex environment with multiple alternatives, the brain must toggle between feeding and non-feeding behaviors that are also relevant for survival. Our results highlight the complexity of this process, describing how—even in the presence of attractive food and in the absence of pain or danger—the brain prioritizes nutritionally maladaptive exercise over food intake.

We identified endogenous HON activity as a regulator of the process that maintains exercise in the presence of an HPF alternative. In our maze paradigm, which examined acute decision-making in a multiple choice scenario, the effect of pharmacological or optogenetic disruption of HON activity on exercise or HPF consumption was greater when both options were available than when only one of these options was available. This context dependence of HON influence suggests that, in the multiple choice maze, HONs acutely implemented TRVE through a valuation mechanism. Importantly, in our experiments, experimental duration was short and fixed, such that committing to the running wheel came at an HPF ‘opportunity cost’ and vice versa. The steep negative relationship between HPF and running wheel use upon orexin receptor blockade (Fig. 4h) suggests an elastic demand^43^ for the two options, whereas the inelastic (that is, much less steep) relationship in the vehicle-injected mice reveals a more fixed demand for HPF independent of running wheel use (Fig. 4h). Therefore, HONs may oppose a demand elasticity-based valuation^43^ between feeding and exercise, thereby promoting TRVE.

Previous studies postulated that HONs regulate eating and locomotion^15,16,30^. We do not think that our new results conflict with these classic findings because of the fundamental differences in study design and intentions. To the best of our knowledge, previous studies did not examine the roles of HONs in acute choice between multiple behavioral alternatives under conditions that limit fatigue or satiation. Previous studies used more isolated contexts than our multiple choice paradigms, and documented the effects of HON manipulations over longer time periods (typically hours or days). Given that HONs affect multiple aspects of energy balance, notably energy expenditure, the chronic effects of HON interference are difficult to interpret because there are well-known causal links between body energy depletion, repletion, fatigue and multiple aspects of behavior, including locomotion^44–47^. Our findings do not rule out that, for example, prolonged HON activation may eventually reduce body energy levels, thus triggering compensatory eating or food-seeking. The profound context dependence of how HON activity is interpreted (Fig. 4) also does not rule out the existence of contexts in which acute HON manipulation alters only eating or only running. Our present results merely indicate that isolated control of eating or running by HONs was not a probable explanation for the TRVE in our multiple choice maze.

Finally, examining rapid neural representations during choice-making in the maze revealed that natural HON dynamics displayed action-specific signatures. A model of these rapid fluctuations indicated that HON activity was positively related to locomotor behaviors and negatively related to HPF licking. When integrated over time, these relationships probably explain the effects of HON loss of function on TRVE. One of the interesting findings that emerged from the analysis of HON dynamics is that, at least in relation to starts and stops of eating or running, HON dynamics were similar in different choice scenarios (Fig. 6f). These data imply that the interpretation of HON signals by downstream circuits, rather than the HON signals themselves, may differ between contexts (as supported by data in Fig. 6b,g). How these differences arise may be related to functional reconfigurations of the multiple downstream targets of HONs in different contexts, in specific ways that remain to be experimentally clarified.

Our findings describe a method and a genetically defined entry point for further study of the biological underpinnings of voluntary exercise in a multiple-alternative environment. This will enable further experiments, aimed at dissecting the roles of specific features of the HON system (projections, cotransmitters, subpopulations) in interacting with the proposed neural drivers of voluntary exercise, such as the dopaminergic and endocannabinoid systems^48,49^, and with neurons and contexts that prioritize other alternatives. Because HONs are present in humans, it is tempting to speculate that our findings may be relevant to humans, although direct tests of this remain to be performed. Such further understanding of adaptive and maladaptive choices relating to exercise may shed light on individual decisions impacting global human health, such as diet-induced or underexercising-induced obesity, or exercise during anorexia.

## Methods

### Animals

All animal experiments followed Swiss Federal Food Safety and Veterinary Office Welfare Ordinance (Animal Welfare Ordinance 455.1, approved by the Zürich Cantonal Veterinary Office). Adult female and male C57BL/6 mice were studied (the sex is noted in Extended Data Table 1). For the HON ablation experiments (Extended Data Fig. 3), we used a previously validated HON-DTR ablation model^35^. Animals were housed in a 12-h reversed light–dark cycle at 22 °C with 55% humidity. All experiments were performed during the dark phase. Animals had ad libitum access to water. To ensure stable motivation, mice were subjected to a mild overnight food restriction (light cycle) before the behavioral experiments, unless stated otherwise. When relevant, cohorts were structured to allow a pseudorandomized crossover design.

### Surgeries and viral vectors

In the fiber photometry experiments, the activity of HONs was measured using a previously validated HON-specific hORX-driven GCaMP6s sensor^35,50^. Briefly, mice were anesthetized using 2–5% isoflurane according to operative analgesia using buprenorphine and site-specific lidocaine. The GCaMP6s calcium indicator was stereotaxically injected unilaterally (randomized) into the lateral hypothalamus (AAV1-hORX-GCaMP6s.hGH, 2 × 10^13^ genome copies (GCs) per ml, Vigene Biosciences). Coordinate injections from bregma were as follows: anteroposterior, −1.35; mediolateral, ±0.90; dorsoventral, −5.70, −5.40 and −5.10, 70 nl at 1 nl s^−1^ per site using a Nanoject III injector). Optic fibers (200-μm diameter, 0.39 numerical aperture fiber with a 1.25-mm ceramic ferrule; Thorlabs) were implanted unilaterally above the injection site in the lateral hypothalamus (anteroposterior, −1.35; mediolateral, ±0.90; dorsoventral, −5.00).

In the optogenetic stimulation experiments, we used the previously developed and validated^23^ ChR driven by the orexin promoter (AAV9-hORX-ChrimsonR-mCherry, 2 × 10^12^ GCs per ml, UZH Viral Vector Facility), which was stereotaxically injected bilaterally into the lateral hypothalamus at the same coordinates and volume as above. In the optogenetic experiments, the control animals were identically fiber-implanted after lateral hypothalamus injection of either an orexin promoter non-opsin virus (tdTomato AAV1-hORX.tdTomato, 1.5 × 10^11^ GCs per ml, ETH Vector and Virus Production) or saline. Optic fibers were implanted bilaterally above the injection site at the same coordinates as for the fiber photometry, but one fiber was implanted 10 degrees mediolaterally to allow space for simultaneous bilateral stimulation. Mice were given postoperative analgesia and allowed to recover for at least 2 weeks before the experiments began.

### Histology

Animals were terminally anesthetized using pentobarbitone and perfused with a sterile PBS solution at pH 7.4 followed by 4% paraformaldehyde in PBS. Brains were removed and then kept in a 4% paraformaldehyde solution overnight, and then in 30% sucrose for another night. Brains were frozen using dry ice and then sectioned at 50 μm with a cryostat. Images were acquired using a fluorescence microscope (Eclipse Ti2, Nikon). When relevant, HONs were identified using staining with goat anti-orexin-A (1:250 dilution, cat. no. sc-8070, Santa Cruz Biotechnology) and donkey anti-goat Alexa Fluor 488 (1:500 dilution, cat. no. A11055, Invitrogen). HON-specific expression of transgenes was confirmed as in our previous studies^23,50^. Similarly, melanin-concentrating hormone neurons were identified using staining with rabbit anti-melanin-concentrating hormone (1:500 dilution, cat. no. H-070-47, Phoenix Pharmaceuticals) and goat anti-rabbit Alexa Fluor 546 (1:500 dilution, cat. no. A-11035, Invitrogen).

### Fiber photometry and modeling

The fiber photometry experiments used a multifiber camera-based photometry system (Doric) using alternating illumination at 405 nm and 465 nm at 20 Hz, with an average power of 70 μW. HON-GCaMP6s emission fluorescence was recorded wherein a 405-nm light-emitting diode was used as an isosbestic control for movement-related artifacts, and 465 nm represented GCaMP6s calcium-dependent HON dynamics. GCaMP6s bleaching was controlled for by fitting and subtracting a triple exponential curve to the full trace. For the following analyses, each trace was either *z*-score-normalized to the entire trace or a percentage Δ*F/F*_0_ was used as specified.

For Fig. 5g, an LMEM was used to predict the *z*-scored GCaMP6s photometry signal. Licking, wheel running speed and maze speed in the *xy* plane were convolved (using a 60-s long decay kernel with a decay rate equivalent to the reported GCaMP6s half-life (1.796 s))^51^ and then *z*-scored along with the isosbestic point (405 nm) to form the fixed effects. Each mouse was a random effect with a free slope with respect to licking, wheel running and running in the *xy* plane of the maze. *Y*_465nm, *ij*_ in the LMEM denotes the predicted *z*-scored response of the *i*th sample from the *j*th mouse given the fitted input variables.

The model is as follows:

$$\begin{array}{c}{y}_{465\mathrm{{nm}}{ij}}={\beta }_{0}+{\beta }_{1}\times {{xy}}_{{ij}}+{\beta }_{2}\times {{\mathrm{wheel}}}_{{ij}}+{\beta }_{3}\times {{\mathrm{licking}}}_{{ij}}+{\beta }_{4}\times {405{\mathrm{nm}}}_{{ij}}\\ +{b1}_{i}\times {{xy}}_{{ij}}+{b2}_{i}\times {{\mathrm{wheel}}}_{ij}+{b3}_{i}\times {\mathrm{{licking}}}_{{ij}}+ {b{4}_{i}\times {405{\mathrm{nm}}}_{{ij}}+\varepsilon}_{{ij}}\end{array}$$

where *β*_0_ is the fixed-effect intercept, *β*_1–4_ are the fixed-effect slopes, *b*1–4_*i*_ are the random-effect slopes and *ε*_*ij*_ is the residual error.

The model was fitted using the lme4 library in R. We assumed that all random effects were normally distributed. *R*^2^ values were computed using Nakagawa’s *R*^2^ for mixed models.

### A free-choice, eight-arm maze

Mice were introduced to an eight-arm arm maze, featuring a central area from which eight identical arms extended outward. In our study, we modified the traditional use of the radial arm maze, originally used by Olton and Samuelson to assess spatial learning and memory^52^. Instead, we adapted the task to capitalize on the innate exploratory behavior of mice, allowing them to naturally engage in various activities and develop their preferences. Unlike traditional maze training paradigms, mice in this task did not require previous training to navigate and explore the maze; they exhibited spontaneous exploration from the initial testing session. Mice were allowed to explore freely for 10 min. This short experimental duration was chosen because it allowed us to efficaciously study the acute, free-choice explorative strategies used by each mouse. Furthermore, in a separate experiment, mice underwent extended sessions lasting 2 h. This was done to evaluate whether they maintained their initial exploratory behavior or altered their preference between HPF and running wheel when provided with more time in the maze. The arms contained different alternatives as follows: a running wheel (Scurry Tethered Mouse Wheel, cat. no. 80840WB, Lafayette Instrument Company); normal laboratory chow (3430 Kliba Nafag); a novel (unfamiliar) same-sex conspecific mouse; a dish of water; a novel (unfamiliar) object; an illuminated ‘light’ arm; and a dark arm. Light and dark arms were insulated with nontransparent plastic to minimize light spillage. A final arm was left empty at the beginning of the experiment but was later fitted out with a custom-built HPF dispenser (cat. no. 161K011, NResearch) with a capacitor-based lick sensor (cat. no. AT42QT-1010, SparkFun Electronics) to gauge consumption. When included, 6 µl of HPF was dispensed for every 10 detected licks. We used milkshake (energy milk strawberry flavor, 0.76 kcal ml^−1^, Emmi AG) as HPF because there is abundant evidence that it is highly palatable and attractive for both mice and humans^53–57^.

To habituate the mice to the maze, on the first day they were placed for 10 min into the empty maze. Then, on the following 7 days, all arm contents (except HPF) were introduced while the position and running wheel activity of mice were recorded using a ceiling-mounted camera. Only the final day was used for the statistical analysis. Proper habituation is crucial to minimize stress and anxiety that could otherwise interfere with the interpretation of task performance.

After the running wheel-only experiments, to avoid food neophobia, mice were acclimated to HPF in their home cages for 1 day. On subsequent days, they were confined to the HPF arm of the apparatus; their preference for the food was assessed by monitoring their food intake, either on reaching 100 rewards, equivalent to 600 µl, or a maximum of 30 min of testing, whichever occurred first. All mice successfully met this threshold within 2 days. The HPF + running wheel experiments were performed in the radial maze. Finally, the wheel arm was closed and mice were assessed once again for place preference in the eight-arm maze without access to the running wheel.

When relevant, vehicle or drug injections and optostimulation were pseudorandomized and staggered across days. Fiber photometry was always recorded when applicable.

### Bout microstructure analysis

Wheel running or licking behaviors were recorded at 500 Hz using a digital I/O device (cat. no. USB-6001, National Instruments) or the digital input of the photometry system detailed above. Traces were binned to 5 Hz and thresholds were defined as 10 cm s^−1^ for running and as any capacitor contact for licking. Bouts were defined as the first incidence above the threshold separated by at least 1 s of non-activity. Bout duration and the amount per bout were quantified as the temporal distance between the start and end of a bout, or the cumulative sum of all behavior during the bout, respectively. For photometry-aligned bouts, this duration was expanded to 4 s of non-activity to allow for a longer duration of the signal to be plotted.

### Simple two-choice maze

The behavior experiments were conducted in a custom made 194 × 181 × 398 mm^3^ acrylic chamber to assess consummatory decision-making between HPF and running wheel. Mice were introduced into the testing chamber where both the running wheel and HPF were accessible in close proximity, aiming to reduce the impact of appetitive place-preference-driven behaviors. On one side of the chamber a running wheel was attached; on the other side, the HPF was delivered in 6-µl increments via the same mechanism as in the eight-arm maze. While performing the task, mice moved freely in the chamber and could either choose the HPF or running wheel for 10 min. All training sessions were conducted in the dark or under red light conditions. Mice were food-deprived for 2 h before behavioral testing to reduce variability in investigatory parameters across animals.

### Pharmacological experiments

A total of 30 mg kg^−1^ (per kg of body weight) of the dual orexin receptor antagonist ALMO (hydrochloride, MedChemExpress), 20 mg kg^−1^ of the orexin receptor-1 antagonist SB-334867 (cat. no. 1960, Tocris Bioscience) or 20 mg kg^−1^ of the orexin receptor-2 antagonist MK-1064 (cat. no. HY-19914, Lucerna Chem) was administered in 2% dimethylsulfoxide and PBS vehicle with 25% 2 hydroxypropyl-β-cyclodextrin. These concentrations of the orexin receptor antagonists were chosen based on previous publications documenting their effects in mice^58–60^. Drug or vehicle solution was administered (pseudorandomly) via intraperitoneal injection 40 min before performing the behavior experiments. Mice had been habituated to intraperitoneal injections of PBS before the experimental day.

### Optogenetic experiments

In the optogenetic experiments, red laser (635 nm, Laserglow) stimulation (7–10 mW at the fiber tip, 20 Hz, 5-ms pulse duration) was applied bilaterally to the lateral hypothalamus for the duration of the experiment. The experiments were performed on mice expressing the excitatory opsin ChR or a mix of tdTomato-expressing and sham-injected animals. Laser ON and laser OFF experiments were performed in a pseudorandom order. No difference was noted between tdTomato-expressing and sham-injected animals, so they were pooled to constitute the control group. Mice had been previously habituated to bilateral optic patch cord tethering before the day of the experiment.

### Data collection, analysis and statistics

Locomotion on the running wheel, as well as licking from the HPF, were recorded at 500 Hz using custom Python scripts and a digital I/O device (NI-DAQmx, National Instruments). Spatial location in the radial maze was recorded using an infrared camera at 5 Hz and then extracted using a custom deep learning network trained on over 10,000 manually labeled frames. Paths were smoothed using a three-sample boxcar filter and then aligned to nine unique regions of interest representing the eight arms of the maze plus the center. Points in which the mouse could not be identified made up less than 0.1% of the data and were linearly interpolated. No statistical methods were used to predetermine sample sizes but our sample sizes are similar to those reported in previous publications^31,50^. Experimenters were blinded to the identity of the optogenetics cohorts. For HON-DTR and the pharmacological injections, data collection and analysis were not performed blinded to the conditions of the experiments. However, maze location data collection was entirely automated using a deep learning network, blinded to mouse identifiers. Raw data processing was performed using Python. Statistical analysis was performed in R or in Python using the Pingouin and SciPy libraries. Data distribution was assumed to be normal but this was not formally tested. No animals or data points were excluded from the analyses. Significance was defined as the following *P* values: *P < 0.05, **P < 0.01, ***P < 0.001. NS represents *P* > 0.05.

### Reporting summary

Further information on research design is available in the Nature Portfolio Reporting Summary linked to this article.

## Online content

Any methods, additional references, Nature Portfolio reporting summaries, source data, extended data, supplementary information, acknowledgements, peer review information; details of author contributions and competing interests; and statements of data and code availability are available at 10.1038/s41593-024-01696-2.

## Supplementary information

Reporting Summary

## Source data

Source Data Fig. 1 Statistical source data.

Source Data Fig. 2 Statistical source data.

Source Data Fig. 3 Statistical source data.

Source Data Fig. 4 Statistical source data.

Source Data Fig. 5 Statistical source data.

Source Data Fig. 6 Statistical source data.

Source Data Extended Data Fig. 1 Statistical source data.

Source Data Extended Data Fig. 2 Statistical source data.

Source Data Extended Data Fig. 3 Statistical source data.

Source Data Extended Data Fig. 4 Statistical source data.

## Figures and Tables

**Extended Data Table 1:**
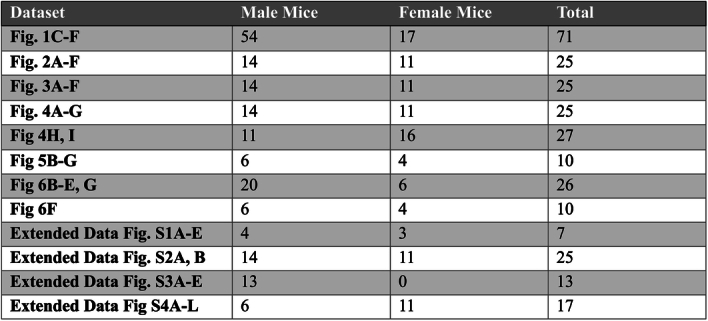
Sex of mice used in all experiments

## Data Availability

Source data can be found at https://osf.io/8dyan/. Source data are provided with this paper.

## Code availability

The code used in the manuscript can be found at https://osf.io/8dyan/.
