# Supplementary material for: Orexin neurons mediate temptation-resistant voluntary exercise
Source: Nat Neurosci. 2024 Aug 6;27(9):1774–82. doi: 10.1038/s41593-024-01696-2 (PMC11374669; doi:10.1038/s41593-024-01696-2)
Supplement: Supplementary file 1 — Reporting Summary [file 41593_2024_1696_MOESM1_ESM.pdf]

nature portfolio

Corresponding author(s): Denis Burdakov
Daria Peleg-Raibstein

Last updated by author(s): May 6, 2024

Reporting Summary

Nature Portfolio wishes to improve the reproducibility of the work that we publish. This form provides structure for consistency and transparency in reporting. For further information on Nature Portfolio policies, see our Editorial Policies and the Editorial Policy Checklist.

Statistics

For all statistical analyses, confirm that the following items are present in the figure legend, table legend, main text, or Methods section.

n/a	Confirmed	
☐	☒	The exact sample size (*n*) for each experimental group/condition, given as a discrete number and unit of measurement
☐	☒	A statement on whether measurements were taken from distinct samples or whether the same sample was measured repeatedly
☐	☒	The statistical test(s) used AND whether they are one- or two-sided<br>*Only common tests should be described solely by name; describe more complex techniques in the Methods section.*
☐	☒	A description of all covariates tested
☐	☒	A description of any assumptions or corrections, such as tests of normality and adjustment for multiple comparisons
☐	☒	A full description of the statistical parameters including central tendency (e.g. means) or other basic estimates (e.g. regression coefficient) AND variation (e.g. standard deviation) or associated estimates of uncertainty (e.g. confidence intervals)
☐	☒	For null hypothesis testing, the test statistic (e.g. *F*, *t*, *r*) with confidence intervals, effect sizes, degrees of freedom and *P* value noted<br>*Give P values as exact values whenever suitable.*
☒	☐	For Bayesian analysis, information on the choice of priors and Markov chain Monte Carlo settings
☒	☐	For hierarchical and complex designs, identification of the appropriate level for tests and full reporting of outcomes
☐	☒	Estimates of effect sizes (e.g. Cohen's *d*, Pearson's *r*), indicating how they were calculated

Our web collection on statistics for biologists contains articles on many of the points above.

Software and code

Policy information about availability of computer code

Data collection	Data collection Python 3.8+, NI-DAQmx 0.7.0, Spinnaker SDK 2.7.0, Doric Neuroscience Studio V5
Data analysis	Python 3.8+, R 4.3, lme4 1.1-31

For manuscripts utilizing custom algorithms or software that are central to the research but not yet described in published literature, software must be made available to editors and reviewers. We strongly encourage code deposition in a community repository (e.g. GitHub). See the Nature Portfolio guidelines for submitting code & software for further information.

Data

Policy information about availability of data

All manuscripts must include a data availability statement. This statement should provide the following information, where applicable:

- Accession codes, unique identifiers, or web links for publicly available datasets
- A description of any restrictions on data availability
- For clinical datasets or third party data, please ensure that the statement adheres to our policy

Preprocessed data and example custom code have been uploaded to an online repository: https://osf.io/8dyan/

Further raw data / metadata are available from corresponding authors upon reasonable request.

Research involving human participants, their data, or biological material

Policy information about studies with human participants or human data. See also policy information about sex, gender (identity/presentation), and sexual orientation and race, ethnicity and racism.

Reporting on sex and gender	No human participants were involved in this study.
Reporting on race, ethnicity, or other socially relevant groupings	No human participants were involved in this study.
Population characteristics	No human participants were involved in this study.
Recruitment	No human participants were involved in this study.
Ethics oversight	No human participants were involved in this study.

Note that full information on the approval of the study protocol must also be provided in the manuscript.

Field-specific reporting

Please select the one below that is the best fit for your research. If you are not sure, read the appropriate sections before making your selection.

☒ Life sciences ☐ Behavioural & social sciences ☐ Ecological, evolutionary & environmental sciences

For a reference copy of the document with all sections, see nature.com/documents/nr-reporting-summary-flat.pdf

Life sciences study design

All studies must disclose on these points even when the disclosure is negative.

Sample size	Sample size was not statistically predetermined, and has instead be chosen such as to be comparable to previous publications: https://www.ncbi.nlm.nih.gov/pmc/articles/PMC4373539/.
Data exclusions	No data was excluded
Replication	Each experiment was replicated across least two independent cohorts
Randomization	Order of vehicle and drug-injections were randomized per-mouse when relevant. For HON-ablated (DTR+) related experiments, WT (DTR-) controls were age and sex-matched litter-mates.
Blinding	Wherever multiple groups of animals (e.g. ORX-DTR) were compared the experimenter was blinded to group identity. Analysis of location in the experiment was not performed manually by humans (rather, a deep-net) therefore further blinding was not required.

Reporting for specific materials, systems and methods

We require information from authors about some types of materials, experimental systems and methods used in many studies. Here, indicate whether each material, system or method listed is relevant to your study. If you are not sure if a list item applies to your research, read the appropriate section before selecting a response.

Materials & experimental systems

n/a	Involved in the study	
☐	☒	Antibodies
☒	☐	Eukaryotic cell lines
☒	☐	Palaeontology and archaeology
☐	☒	Animals and other organisms
☒	☐	Clinical data
☒	☐	Dual use research of concern
☒	☐	Plants

Methods

n/a	Involved in the study	
☒	☐	ChIP-seq
☒	☐	Flow cytometry
☒	☐	MRI-based neuroimaging

Antibodies

Antibodies used	Primary antibodies:<br>Goat anti-orexin A (Santa Cruz, sc-0870, 1:250 dilution, LOT H2916)

	Rabbit anti-MCH (Pheonix Pharamceuticals, H-070-47, 1:500 dilution, LOT 01477-4)<br><br>Secondary antibodies:<br>Donkey anti-goat Alexa-488 (Invitrogen, A11055, 1:500 dilution, LOT 2059218)<br>Goat anti-rabbit Alexa-546 (Invitrogen, A-11035, 1:500 dilution, LOT 2129899)
Validation	Antibodies used in this study were purchased from commercially available sources and were validated by the manufacturer.<br><br>For goat anti-orexin A: https://www.scbt.com/p/orexin-a-antibody-c-19<br><br>For rabbit anti-MCH: https://phoenixpeptide.com/products/view/Antibodies/H-070-47<br><br>For donkey anti-goat Alexa-488: https://www.thermofisher.com/antibody/product/Donkey-anti-Goat-IgG-H-L-Cross-Adsorbed-Secondary-Antibody-Polyclonal/A-11055<br><br>For goat anti-rabbit Alexa-546: https://www.thermofisher.com/antibody/product/Goat-anti-Rabbit-IgG-H-L-Highly-Cross-Adsorbed-Secondary-Antibody-Polyclonal/A-11035

Animals and other research organisms

Policy information about studies involving animals; ARRIVE guidelines recommended for reporting animal research, and Sex and Gender in Research

Laboratory animals	This study used adult mice (>10 weeks). Strains: C57BL/6 and ORX-DTR (C57BL/6-background). Mice were co-housed in groups of 2-5 animals of the same sex. Housing conditions were a 12-hour reversed light/dark cycle at 22°C with 55% humidity.
Wild animals	This study did not involve wild animals.
Reporting on sex	Both sexes were used in every experiment (except for Supp. Fig. S3; DTR cohorts where only male mice were used). Statistical comparisons including sex as an effect are provided the figure legend of Fig. 4 and in Supp. Fig. 2. Broadly, we found no significant sex differences in our experiments, so both sexes were pooled. A table detailing the exact number of male and female mice used in every plot is found in Supp. Table. 1.
Field-collected samples	This study did not involve field-collected samples.
Ethics oversight	All animal experiments were performed in accordance with the Animal Welfare Ordinance (TSchV 455.1) of the Swiss Federal Food Safety and Veterinary Office, and approved by the Zürich Cantonal Veterinary Office.

Note that full information on the approval of the study protocol must also be provided in the manuscript.

Plants

Seed stocks	This study did not involve plants.
Novel plant genotypes	This study did not involve plants.
Authentication	This study did not involve plants.
