# Supplementary material for: Orexin neurons mediate temptation-resistant voluntary exercise
Source: Nat Neurosci. 2024 Aug 6;27(9):1774–82. doi: 10.1038/s41593-024-01696-2 (PMC11374669; doi:10.1038/s41593-024-01696-2)
Supplement: Supplementary file 6 — Statistical source data. [file 41593_2024_1696_MOESM6_ESM.zip › LMEM_Photometry.html]

LMEM\_Photometry


# LMEM\_Photometry

#### Alexander L Tesmer

#### 2024-04-29

## Imports

Import required libraries, and read the datafile

```
library("readxl")
library("lme4")
library("lmerTest")
library("performance")
df = read_excel("P:/Alexander/StarMaze/Modeling_Input.xlsx")
```

## Preprocessing

Ensure that R understands which variables are numeric, or
categorical. The dataframe should already be normalized to mean and
standard deviation (on a per-mouse basis).

```
df$Sig = as.numeric(df$Sig) # 470 nm GCaMP6s from HONs
df$Ref = as.numeric(df$Ref) # 405 nm isosbestic control from HONs

df$Disp = as.numeric(df$Disp) # GCaMP6s-kernel convolved speed in the maze
df$Vol = as.numeric(df$Vol) # GCaMP6s-kernel convolved HPF-licking
df$SpeedW = as.numeric(df$SpeedW) # GCaMP6s-kernel convolved speed on the wheel

df$Mouse = as.factor(df$Mouse) # Mouse ID
```

## Modeling

Fit a linear mixed-effects model using the package lme4 and wrapper
lmerTest. Note we have chosen not to fit random-intercepts.

```
# Fit the model
m1 <- lmer(Sig ~ 1 + Disp + Vol + SpeedW + Ref + (0+Disp|Mouse)+(0+Vol|Mouse)+(0+SpeedW|Mouse)+(0+Ref|Mouse),
           control=lmerControl(optimizer="bobyqa"),data=df,REML=TRUE)
summary(m1)
```

```
## Linear mixed model fit by REML. t-tests use Satterthwaite's method [
## lmerModLmerTest]
## Formula: Sig ~ 1 + Disp + Vol + SpeedW + Ref + (0 + Disp | Mouse) + (0 +  
##     Vol | Mouse) + (0 + SpeedW | Mouse) + (0 + Ref | Mouse)
##    Data: df
## Control: lmerControl(optimizer = "bobyqa")
## 
## REML criterion at convergence: 58452.6
## 
## Scaled residuals: 
##     Min      1Q  Median      3Q     Max 
## -3.8034 -0.5537 -0.0230  0.5710  4.9290 
## 
## Random effects:
##  Groups   Name   Variance Std.Dev.
##  Mouse    Disp   0.02233  0.1494  
##  Mouse.1  Vol    0.05699  0.2387  
##  Mouse.2  SpeedW 0.03492  0.1869  
##  Mouse.3  Ref    0.03608  0.1900  
##  Residual        0.50803  0.7128  
## Number of obs: 26950, groups:  Mouse, 10
## 
## Fixed effects:
##               Estimate Std. Error         df t value Pr(>|t|)    
## (Intercept)  2.196e-04  4.342e-03  2.691e+04   0.051 0.959654    
## Disp         3.089e-01  4.753e-02  9.007e+00   6.500 0.000111 ***
## Vol         -3.509e-01  7.569e-02  8.992e+00  -4.636 0.001229 ** 
## SpeedW       2.005e-01  5.933e-02  9.010e+00   3.379 0.008125 ** 
## Ref         -5.263e-02  6.026e-02  9.010e+00  -0.873 0.405179    
## ---
## Signif. codes:  0 '***' 0.001 '**' 0.01 '*' 0.05 '.' 0.1 ' ' 1
## 
## Correlation of Fixed Effects:
##        (Intr) Disp   Vol    SpeedW
## Disp    0.000                     
## Vol     0.000  0.003              
## SpeedW  0.000  0.004  0.003       
## Ref     0.000  0.001 -0.001  0.000
```

```
# Diagnostics: print R2 using Nakagawa's method
print(r2_nakagawa(m1),digits=6)
```

```
## # R2 for Mixed Models
## 
##   Conditional R2: 0.418260
##      Marginal R2: 0.392671
```

```
# Compute confidence intervals (this takes a while)
confint(m1)
```

```
##                    2.5 %       97.5 %
## .sig01       0.095910609  0.237429982
## .sig02       0.153823186  0.378738608
## .sig03       0.120236650  0.296616487
## .sig04       0.122434320  0.301505074
## .sigma       0.706768538  0.718812302
## (Intercept) -0.008290076  0.008729626
## Disp         0.211402083  0.406462778
## Vol         -0.506331984 -0.195604265
## SpeedW       0.078755997  0.322293797
## Ref         -0.176381410  0.071081354
```

Note the generation of p-values from a linear mixed-effects model is
controversial, and should be interpreted with some caution. See https://stat.ethz.ch/pipermail/r-help/2006-May/094765.html
by Douglas Bates for discussion. Similarly, Nakagawa’s R2 may be
controversial.
